# Feasibility and Effectiveness of an Adolescent Glass‐Analysis Reading Intervention Delivered Online by Lay Tutors

**DOI:** 10.1002/dys.70046

**Published:** 2026-08-19

**Authors:** Corrine Durisko, Kailee Lear, Isabel Beck, Cheryl Sandora, Julie A. Fiez

**Affiliations:** ^1^ Learning Research and Development Center University of Pittsburgh Pittsburgh Pennsylvania USA; ^2^ Department of Psychology University of Pittsburgh Pittsburgh Pennsylvania USA; ^3^ School of Education University of Pittsburgh Pittsburgh Pennsylvania USA; ^4^ Center for the Neural Basis of Cognition University of Pittsburgh Pittsburgh Pennsylvania USA; ^5^ Department of Communication Science and Disorders University of Pittsburgh Pittsburgh Pennsylvania USA

**Keywords:** analytic phonics, decoding, dyslexia, fluency, literacy

## Abstract

Many adolescents do not have the basic reading skills needed to successfully comprehend increasingly complex academic texts, and the COVID‐19 pandemic has further disrupted reading achievement. These concerns motivate the present pilot study, which adapts the Glass‐Analysis programme, a theoretically distinctive analytic phonics intervention, for online delivery by lay tutors. Fifth‐ and sixth‐grade students (*N* = 34) with poor reading skills were alternately assigned to an intervention or waitlist control group. Intervention participants completed individualised sessions over 11–19 weeks. The Woodcock Reading Mastery Test (WRMT‐III) and Test of Word Reading Efficiency (TOWRE‐2) were used to measure changes in reading skills. Tutors reached high fidelity at the end of training; technological barriers were minimal, and tutor and participant retention was high. Moderate effect size differences between the intervention and waitlist control group were observed for measures of decoding and rapid automatised naming, but the study was designed with low statistical power and none of the observed group differences were significant. We conclude the Glass‐Analysis programme holds promise as a scalable and accessible reading intervention for adolescent struggling readers and that future work to more definitively test its effectiveness is warranted.

## Key Points

- The Glass‐Analysis programme uses a distinctive analytic phonics approach to build decoding fluency.
- It is feasible to deliver the Glass‐Analysis programme online using lay tutors.
- There is promising evidence that the Glass‐Analysis programme is effective for adolescent struggling readers.
- The Glass‐Analysis programme and tutor training materials are available at relatively low‐cost to educators and the public.
- The Glass‐Analysis programme can be flexibly delivered to individuals or small groups, within or beyond schools and in‐person or online.

## 1 Introduction

The most recent report on fourth‐grade reading achievement reveals continuing declines since the COVID‐19 pandemic, with overall scores now approaching their lowest level in more than 30 years (National Center for Education Statistics 2024). As adolescents who are poor readers progress into middle school, they are likely to have few opportunities to improve their basic skills (National Reading Panel 2000; Snow 2010), which places them at high risk for continued reading struggles that pervasively impact their academic success and life trajectory (Bigozzi et al. 2017; Hock et al. 2009; Wang et al. 2019). This creates an urgent need to identify effective and scalable interventions for adolescents with poor basic reading skills. Here, we respond to this urgent need by conducting a pilot study involving 34 fifth‐ and sixth‐grade struggling readers assigned to either a reading intervention or a waitlist control group. The study has two interrelated goals: the first is to investigate the potential effectiveness of a promising but underexplored analytic phonics intervention, and the second is to evaluate the feasibility of delivering this intervention online using lay tutors. Positive outcomes would motivate and inform future work with adequate metrics and statistical power to detect improvements in reading skill and assess implementation fidelity.

More specifically, the study uses the Glass‐Analysis reading intervention (Glass 1973; Glass and Glass 1990). Glass‐Analysis involves an analytic phonics approach that centres around multi‐letter orthographic clusters. Each cluster is taught using a set of service words that contain the orthographic cluster. The tutor presents each service word individually, pronouncing it aloud and then using a formulaic script that requires the student to attend to the letters in each cluster and their relationship to the spelling and phonology of the service word. After a set of service words has been analysed, the student reads aloud sentences that include words containing the orthographic cluster. Glass‐Analysis is theorised to shape visual‐speech decoding habits so that a reader's visual attention is more automatically drawn to orthographic clusters and their relationship to a surrounding whole word context. Over time, this builds stimulus (orthographic clusters) and response (corresponding phonology) bonds. As the decoding habits and bonds strengthen, this facilitates the implicit and fluent decoding of unfamiliar words (Glass 1973; Miccinati 1981).

Glass‐Analysis (Glass 1973; Glass and Glass 1990) differs from more commonly used synthetic phonics approaches, which provide direct instruction in grapheme‐phoneme correspondences and practice using this knowledge (Beck and Beck 2013). While synthetic phonics approaches are highly effective at building decoding skill in most students, they involve attentional, phonemic analysis and working memory component skills that are difficult for many poor readers (Eden et al. 1996; Farah et al. 1996; Share 2021; Shaywitz and Shaywitz 2008; van der Kleij et al. 2017). Glass‐Analysis might therefore be well suited for adolescent readers who have acquired basic grapheme‐phoneme knowledge and have some ability to decode unfamiliar words but still struggle with fluent (fast and accurate) decoding.

Crucially, Glass‐Analysis (Glass 1973; Glass and Glass 1990) has received relatively little attention from reading researchers. Three studies appeared as doctoral dissertations and involved small samples (Ns of 8–95) of children in elementary grades (Bernosky 1999; Searfoss 1973; Walsh 1990); one appeared as a peer‐reviewed publication and involved a small sample (*N* = 21) of high school participants (Penney 2002). Bernosky (1999) and Penney (2002) found evidence of greater reading gains for a Glass‐Analysis programme as compared to a control group. Searfoss (1973) and Walsh (1990) compared Glass‐Analysis to alternative interventions and did not observe significant differences. The available evidence therefore suggests the Glass‐Analysis programme is beneficial, but it may not be superior to other reading interventions for younger readers.

Another intriguing aspect of Glass‐Analysis is the relative simplicity of its implementation. The materials consist of single words and sentences, and tutors systematically progress through these materials using a highly structured script. This suggests the intervention could be readily adapted to a digital form suitable for online delivery by lay tutors. These pragmatic aspects of Glass‐Analysis could make it an attractive and relatively low‐cost intervention option for schools and reading educators, as well as parents and members of youth‐focused community organisations. Given the critical need to improve adolescent reading achievement, the study design addresses the viability of implementing Glass‐Analysis online using lay tutors, while simultaneously evaluating the efficacy of Glass‐Analysis and building knowledge about the intervention's scalability and accessibility.

## 2 Methods

### 2.1 Participants

Participants were recruited through flyers and email distribution lists maintained by the University and local schools. Determination of study interest and eligibility was conducted by a phone screening with a parent or legal guardian of the prospective participant. Inclusion criteria included rising or current fifth‐ or sixth‐grade student in a public, private or homeschool setting and one or more of the following guardian‐reported indicators of poor reading skill: a dyslexia diagnosis by a clinical or educational professional, an individualised education plan to improve reading, a below‐basic reading score on the Pennsylvania System of School Assessment test, or probable dyslexia based on a validated dyslexia screening questionnaire (Willcutt et al. 2011). Exclusionary criteria were non‐fluent English language proficiency, a diagnosed psychiatric illness, vision or hearing impairment, major medical problems (e.g., cancer), or history of developmental disability other than sensory processing disorder, dysgraphia or attention‐deficit‐hyperactivity (ADHD) disorder. Participants (*N* = 34, 17 male and 17 female) were assigned in alternating order to an intervention or waitlist control group. Participants received $105.00 for completing pretest and post‐test assessments of their reading ability. All procedures were compliant with informed consent and human subjects research practices overseen by the University Institutional Review Board.

### 2.2 Tutors

Tutors were recruited from the University undergraduate student body and were paid $15 hourly. Tutors completed all required institutional certifications for research involving human subjects and state clearances for working with minors. Then, they completed training on how to deliver the Glass‐Analysis (Glass and Glass 1990) intervention. This training was divided into four modules requiring 12–15 h of independent study over a two‐week period including several quizzes to test their understanding. The modules primarily focused on specifics of the intervention, with some additional content on professionalism and cultural sensitivity. The training culminated in a final practice session and fidelity check conducted by an experienced member of the research team to certify proficiency in intervention administration. Following training, tutors participated in one‐hour weekly group meetings that were led by members of the research team and which provided an opportunity for ongoing support and guidance. Tutors worked individually with their assigned students to implement the intervention using an intervention guide that provided detailed information on the printed words and associated script to be used for each tutoring session. Tutors consulted with their intervention participants and their guardians to schedule the intervention sessions.

### 2.3 Glass‐Analysis Programme Materials and Implementation

The intervention was based on the instructor's guide for the Glass‐Analysis programme (Glass and Glass 1990) and printed versions of the intervention materials (available from Easier to Learn, South Orange, NJ, USA). These materials are organised into 119 orthographic clusters. For each cluster there is a set of 16 service words, each containing the target cluster, and typically eight sentences that incorporate a high number of words containing the cluster. With permission, we created a corresponding set of modernised digital materials, replacing some service words judged to be archaic and rewriting most of the sentences. The resulting materials for clusters 1–90 were incorporated into a PowerPoint slide deck. For each cluster, we created one slide for each service word and two slides with the sentences.

**FIGURE 1:**
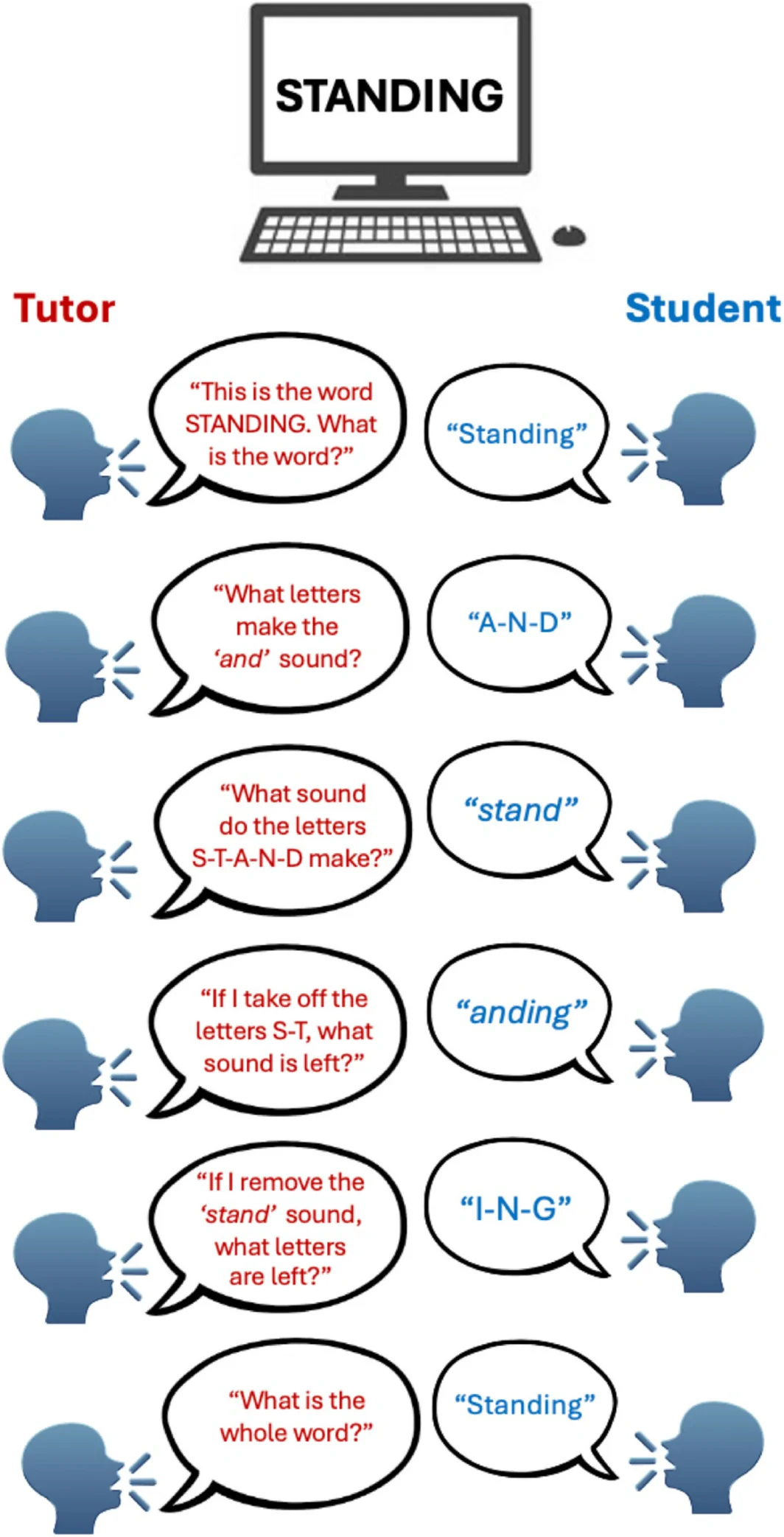
Tutor script for the cluster ‘AND’ with service word (standing). An example cluster sentence is ‘Sandy sings while she stands on her hands’.

The 90 clusters used for the intervention were divided into 30 sessions. To administer each session, the tutor selected 8–10 service words per cluster, and for each word implemented the 6‐step Glass‐Analysis script (Figure 1). Students ended the session by reading the sentences for each cluster taught that day, with the tutor assisting to maintain fluency and accurate decoding of words that contained a trained cluster.

### 2.4 Pre/Post Assessments

Participants completed two online behavioural assessment sessions scheduled up to 2 weeks before (pretest) and after (post‐test) the completion of the intervention (or a yoked duration for those in the waitlist control group). These sessions were conducted by a research team member blind to participants' group assignment. The Woodcock Reading Mastery Test‐III (WRMT‐III) (Woodcock 2011) was administered to all participants during the pretest (using Form A) and post‐test (using Form B), with the exception of two participants who withdrew from the study without completing the post‐test. Six months into the study, we added the Test of Oral Word Reading Efficiency‐Second Edition (TOWRE‐2) (Torgesen et al. 2012) to provide a timed measure of word and pseudoword reading, with Form A used for both the pretest and post‐test sessions. Raw scores on each subtest of the WRMT‐III and TOWRE‐2 were determined based on the scoring instructions and then converted to standardised scores based on the grade (WRMT‐III) and age (TOWRE‐2) of participants at administration of the pretest.

## 3 Results

All control participants completed the pre/post‐test sessions. Early in our study, two intervention participants withdrew after less than five intervention sessions due to scheduling conflicts. Because they received so little of the intervention, we did not invite them to complete the post‐test. After the withdrawal of these two participants, we modified our recruitment script to more fully explain the intervention time commitment and scheduling expectations. Post‐test data were collected from the remaining 15 intervention participants, 13 of whom completed the full training and two of whom ended training before completing all 90 clusters. In one case, the participant completed 67 clusters and then was advised by their school to end training to avoid burnout in association with starting an intensive school‐based reading intervention. In a second case, the participant was unable to regularly sustain our requested scheduling pace and so tutoring was discontinued after 6 months, at which point the participant had completed 60 clusters.

Our inclusion/exclusion criteria were broad, and as expected participants came from varied backgrounds (see Table 1 for demographic and school history information for the study participants). Our recruitment procedures were effective at identifying struggling readers, though considerable individual differences were observed in pretest reading skill. For the 32 participants who completed both the pre and post‐test sessions, the mean standardised WRMT‐III total reading score for the pretest assessment was 79.22 ± 11.37 SD; importantly, while the mean score of the intervention group was higher than the control group (80.07 ± 10.46 SD vs. 78.47 ± 12.39 SD), this difference was non‐significant (*t*(1,31) ≤ 1.50, *p* ≥ 0.14). Individual WRTM‐III total reading scores ranged from 57 (< 1st percentile) to 95 (37th percentile). Nearly all (29/32) participants scored in the below‐average range (< 16th percentile) on at least one composite reading measure (WRMT‐II Basic Reading, WRMT‐II Comprehension, and/or TOWRE‐2 Total Reading Efficiency).

**TABLE 1:** Demographic information for the intervention and waitlist control groups.

	Grade		Gender		Race/ethnicity		Handedness		School type	
Glass intervention	Fifth	10	M	6	White	5	RH	8	Public	6
					Black	3			Private	2
			F	4	More than one race	1*	LH	2	Specialised charter	2
					Prefer not to answer	1			Homeschool	0
	Sixth	7	M	0	White	5	RH	7	Public	4
					Black	2			Private	1
			F	7	More than one race	0	LH	0	Specialised charter	1
					Prefer not to answer	0			Homeschool	1
Wait list control	Fifth	13	M	9	White	10	RH	11	Public	9
					Black	2			Private	0
			F	4	More than one race	0	LH	2	Specialised charter	3
					Prefer not to answer	1			Homeschool	1
	Sixth	4	M	2	White	4	RH	4	Public	2
					Black	0			Private	0
			F	2	More than one race	0	LH	0	Specialised charter	2
					Prefer not to answer	0			Homeschool	0

*Note:* *indicates a significant *t*‐test for pre vs posttest in that group (intervention or control).

All tutors completed the training protocol and successfully attained high fidelity implementation based on review of a final practice session by a member of the research team. Overall, we had a 100% tutor retention rate and most tutors remained with their assigned student(s) until they completed the intervention; however, in a few cases there were changes in schedule availability (such as a change in the academic term or a participant's new extracurricular activity) that led to a change in the tutor‐student pairing. We offered mobile devices and internet hotspots to all participants as part of the screening process. Only one of the 34 participants requested the offered technology. On average, participants completed three sessions per week, each 45–60 min in length, with the overall intervention extending over 11–19 weeks depending upon the pace and scheduling constraints of the participant. None of the tutors or participants reported regular difficulties with the quality or format of the online intervention.

The results of the pre/post‐test reading assessments are summarised in Table 2. Paired one‐sample, one‐tailed *t*‐tests were used to test for score gains (post‐test vs. pretest) for each measure within each group (intervention, waitlist control). In the Glass‐Analysis intervention group we observed significant improvements for the WRMT‐III Rapid Automatized Naming, WRMT‐III Word ID and TOWRE‐2 Phonemic Decoding Efficiency subtests, *ps* < 0.05. In the waitlist control group, we did not observe significant improvements for any of the administered subtests. Unpaired two‐sample, one‐tailed *t*‐tests were used to directly test whether the score gains (post‐test minus pretest score) were larger for the intervention as compared to the control group. We observed moderate effect sizes for the TOWRE‐2 phonemic decoding efficiency and WRMT‐III rapid automatised naming subtests, and smaller but still promising effect sizes for the WRMT‐III Word Attack and Passage Comprehension subtests. Consistent with past findings involving systematic phonics interventions, effects for the WRMT‐III and TOWRE‐2 subtests involving word naming were small, likely because they include many words with inconsistent pronunciations and spelling patterns not targeted by the Glass‐Analysis intervention (National Reading Panel 2000). We expected larger effects for oral reading fluency and believe this may reflect participant frustration since the WRMT‐III protocol begins with grade‐level passages and only steps down to easier passages when a fluency criterion is not met.

**TABLE 2:** Data represent the mean and standard errors of change scores (post‐test minus pretest) for both the intervention and control groups.

	Difference scores					
	Reading tests	Intervention	Control	Intervention vs. control		
		*M* (SE)	*M* (SE)	*t*	*p*	Cohen's *d*
WRMT‐III reading readiness	Letter identification	0.00 (0.00)	0.00 (0.00)	n/a	n/a	n/a
	Phonological awareness	0.87 (0.66)	0.94 (0.56)	−0.09	0.47	−0.03
	Rapid automatised naming	3.53 (1.60)*	−0.12 (1.60)	1.61	0.06	0.57
WRMT‐III basic skills	Word ID	3.60 (1.90)*	3.06 (2.08)	0.19	0.43	0.07
	Word attack	0.20 (3.12)	−3.47 (2.25)	0.97	0.17	0.34
WRMT‐III reading comprehension	Word comprehension	−2.47 (2.44)	2.41 (2.41)	−1.41	0.92	−0.50
	Passage comprehension	5.80 (2.41)	2.65 (2.71)	0.73	0.24	0.26
	Oral reading fluency	−0.60 (1.76)	−2.00 (1.86)	0.54	0.30	0.19
TOWRE‐2	Sight word efficiency	2.20 (2.15)	4.86 (1.37)	−1.09	0.86	−0.45
	Phonemic decoding efficiency	4.60 (1.33)*	1.71 (1.44)	1.41	0.09	0.58

*Note:* These changes are based on standardised scores from the Woodcock Reading Mastery Test (WRMT‐III) and the Test of Oral Word Reading Efficiency (TOWRE‐2), with the exception of the WRMT‐III Reading Readiness tasks. Because these are only normed for Kindergarten, raw scores were used for these specific measures. Final columns provide *t* value, *p* value, and effect size measures for an unpaired, one‐tailed *t*‐test comparing the test score gains (post‐test minus pretest) for the intervention versus waitlist control group.

* Significant positive change (*p* < 0.05) based on a one‐sample, one‐tailed test comparing pre versus post‐test scores.

## 4 Conclusions

The growing number of adolescents with poor reading skills motivated this pilot study, which investigated the potential effectiveness and scalability of the Glass‐Analysis programme (Glass 1973; Glass and Glass 1990). The study developed modernised digital materials and a structured protocol for training lay tutors in its online implementation. Then, a broad sample of 34 fifth‐ and sixth‐grade adolescents with a history of reading difficulty was recruited on a rolling basis, with participants assigned in alternating order to a Glass‐Analysis intervention or a waitlist control group. Standardised assessments of reading skill were administered before and after the intervention (or a similar waitlist duration) to evaluate changes in reading skill. As detailed below, the findings provide promising evidence that Glass‐Analysis may be an effective intervention for adolescents who are struggling readers and that it can be successfully delivered by lay tutors online.

Glass‐Analysis (Glass 1973; Glass and Glass 1990) uses a distinctive analytic phonics approach to strengthen visual analysis habits and orthographic‐phonological bonds. It is thought to improve implicit decoding and thereby produces gains in decoding fluency and sentence comprehension (Glass 1973; Miccinati 1981). Our findings are consistent with this theoretical premise, as the intervention group showed moderate improvement, as compared to the waitlist control group, on measures of rapid automatised naming and pseudoword decoding. Smaller but still noteworthy effects were observed for measures of word identification, passage comprehension and oral reading fluency. While none of these results reached significance, the observed effect sizes are in line with those observed for adolescent reading intervention programmes with demonstrated track records of effectiveness (Vaughn et al. 2022). Moreover, the findings are similar to those from a prior study involving high school poor readers, which also observed improvements in word identification, pseudoword reading and passage comprehension (Penney 2002).

The results further suggest the Glass‐Analysis programme (Glass 1973; Glass and Glass 1990) has high potential scalability and accessibility. The digital materials are available to schools and the public at low‐cost; the tutor training protocol that we developed can be completed in less than 15 h, and our findings complement past studies that involved in‐person, small group intervention delivery by educational professionals. More specifically, we observed that lay tutors demonstrated a high level of fidelity at the end of the training protocol, few technology obstacles were encountered in delivering the intervention, and retention of tutors and participants was high. It should be noted that tutor fidelity or tutor–tutee interpersonal dynamics were not evaluated during the actual delivery of the intervention, and so it is possible that these unmeasured factors may have contributed to individual variability in intervention outcomes.

It is important to caution that this was a pilot study with a small sample size and pragmatic constraints on the experimental design. For instance, to allow for a more even scheduling of tutors, we adopted an alternating rather than randomised participant assignment, and we relied upon guardian‐provided information to determine study eligibility. To address the limitations of this work, future studies should utilise a participant sample with sufficient power to reliably detect small effect sizes and factors that may impact treatment outcomes, poor reading skill should be assessed through objective measures prior to study enrollment, and participants should be randomly assignment to study groups. Additionally, although the WRMT‐III and TOWRE‐2 are widely used as outcome measures in reading intervention studies, they use progressively more difficult stimuli and administration protocols that may make it difficult to detect improvements in decoding the types of words adolescent readers tend to encounter. Standardised assessments that focus on assessing fluency using decodable, multisyllabic words and grade‐level text may yield more robust results. For the intervention itself, concerns about the overall length of our intervention, led to the exclusion of the most difficult clusters that comprise the ‘completers’ level of the Glass‐Analysis clusters, and due to pragmatic issues, the intervention was delivered only 2–3 times per week. Larger intervention gains might be achieved by extending the intervention to incorporate the completers level or alternatively starting the intervention at a more advanced level, and by providing more intervention sessions per week. Additionally, consideration should be given to data acquisition strategies that would allow tutor‐related factors in intervention outcomes to be examined. Finally, it is important to emphasise that our study design used a waitlist control group. Consequently, we cannot rule out the possibility that intervention gains resulted from general aspects of the intervention experience (e.g., positive tutor‐tutee interactions that improved participant's academic self‐confidence or motivation), and our findings provide no direct evidence of the effectiveness of the Glass‐Analysis programme over other types of reading interventions. Future work should consider including an active control condition that has a similar format and tutor structure but does not focus on building decoding skill, as well as an alternative treatment arm that uses a different approach for building decoding skill (e.g., a high‐quality synthetic phonics approach (Beck and Beck 2013)).

In sum, the current study is an important addition to the promising but sparse literature involving the Glass‐Analysis programme (Glass 1973; Glass and Glass 1990). We developed training materials and procedures that allowed lay tutors to successfully deliver the intervention online, and we observed gains on measures of reading skill consistent with the intervention's theoretical mechanism of action. We conclude that the Glass‐Analysis programme deserves renewed attention due to its potential for expanding intervention options and access for the many adolescents who are struggling readers, and we recommend future work with a larger sample size and a more rigorous experimental design so that the effectiveness of Glass‐Analysis can be more definitively established.

## Funding

The work was supported from the Learning Research and Development Center Internal Awards Program.

## Ethics Statement

All participants provided informed consent, and the research was conducted with the approval of the University of Pittsburgh Institutional Review Board.

## Conflicts of Interest

The authors declare no conflicts of interest.

## Data Availability

The data that support the findings of this study are available from the corresponding author upon reasonable request.
